# Life-threatening disseminated enterovirus infection during combined rituximab and ibrutinib maintenance treatment for mantle cell lymphoma: a case report

**DOI:** 10.1186/s13256-020-02457-y

**Published:** 2020-08-28

**Authors:** Maximilian Higer, Denis Cana, Juergen Podlech, Simin Schadmand-Fischer, Andreas Schwarting, Daniel Teschner, Matthias Theobald, Thomas Wölfel, Georg Hess

**Affiliations:** 1 Department of Hematology, Medical Oncology & Pneumology, University Medical Center of the Johannes Gutenberg-University, Langenbeckstrasse 1, D-55131 Mainz, Germany; 2 Division of Neuropathology, University Medical Center of the Johannes Gutenberg-University, Mainz, Germany; 3 Institute for Virology, University Medical Center of the Johannes Gutenberg-University, Mainz, Germany; 4 Department of Diagnostic and Interventional Radiology, University Medical Center of the Johannes Gutenberg-University, Mainz, Germany; 5 Department of Internal Medicine I (Gastroenterology, Hepatology, Nephrology, Rheumatology, Infectiology and Immunology), University Medical Center of the Johannes Gutenberg-University, Mainz, Germany

**Keywords:** Non-Hodgkin lymphoma, B lymphocytes, Rituximab, Enterovirus, Ibrutinib, Myositis

## Abstract

**Background:**

Rituximab is a well-established component of treatment regimens for B-cell non-Hodgkin lymphoma. Rituximab binds the CD20 antigen on the surface of B lymphocytes, causing an enhanced clearance of malignant and benign B cells. Thus, rituximab leads to depletion of normal B lymphocytes as well, which can cause substantial immunodeficiency. Ibrutinib inhibits the Bruton tyrosine kinase and thereby B-cell activity. It is used for the treatment of different B-lymphocyte malignancies, such as mantle cell lymphoma. Recently, the combination of both drugs has been tested in various clinical scenarios.

**Case presentation:**

We present a case of disseminated enterovirus infection resulting from combined rituximab and ibrutinib maintenance treatment in a 57-year-old Caucasian patient. with mantle cell lymphoma. Initially presenting with myositis symptoms, further diagnostic investigation revealed myocarditis, enteritis, myeloencephalitis, and hepatitis. These organ manifestations led to potentially life-threatening complications such as rhabdomyolysis, delirium, and heart rhythm disturbances. After treatment with high-dose intravenous immunoglobulins, virus clearance was achieved and organ functions could be restored.

**Conclusions:**

This case emphasizes the risk of combined therapy with rituximab/ibrutinib for severe immune-related side effects with the necessity of continuous patient monitoring. High-dose intravenous therapy should be considered as treatment for severe enterovirus infection. In severe enterovirus infections, we recommend subtyping for the development of efficient preventive and therapeutic strategies.

## Background

Mantle cell lymphoma (MCL) frequently presents with an aggressive disease course. In consequence, intensive sequential treatment with chemoimmunotherapy induction, high-dose therapy consolidation, and rituximab maintenance has been established as the standard of care for eligible patients; however, patients continue to relapse. Current clinical research strategies test the combination of rituximab and ibrutinib, an inhibitor of Bruton tyrosine kinase (BTK) interacting with the B-cell receptor pathway, in induction and maintenance treatment for MCL. Until now, the toxicity of the long-term combination of these agents has not been well defined. Long-term use of rituximab is usually well tolerated. However, some patients experience sequelae of long-term lymphodepletion, such as reactivation of viruses, including hepatitis B. Ibrutinib has been associated with reduction of humoral immunity and subsequent infections. Moreover, in combination with R-CHOP (rituximab, cyclophosphamide, doxorubicin hydrochloride [hydroxydaunomycin], vincristine sulfate [oncovin], prednisone), an increased toxicity has been noted [1, 2]. In this report, we describe a life-threatening enterovirus infection involving multiple organ systems in a patient with MCL during combined rituximab and ibrutinib maintenance therapy.

Enteroviruses are small ribonucleic acid (RNA) viruses and belong to the *Picornaviridae* family. In immunocompetent individuals, enterovirus infections are usually asymptomatic. If symptoms occur, they usually resemble signs of “common cold” or gastroenteritis. However, even myocarditis, exanthema, encephalomyelitis, or acute paralysis can arise, depending on the virus subtype and immune status of the patient [3]. Moreover, patients with hereditary or acquired B-cell defects may be at risk for persistent, in some cases even fatal, infection [4].

## Case presentation

Our patient was a 57-year-old Caucasian man who was diagnosed in July 2017 with MCL stage cS4a (bone marrow and abdominal, cervical and axillary lymph node involvement) and a high-risk Mantle Cell Lymphoma International Prognostic Index score. Besides controlled arterial hypertension and mild neuropathy, the patient had no significant comorbidities. Within a clinical trial, he was treated with induction chemoimmunotherapy of alternating R-CHOP/R-DHAP (rituximab, dexamethasone, cytarabine, cisplatin), resulting in a complete remission. From February 2018, the patient received maintenance therapy with ibrutinib (560 mg once daily) and rituximab (1400 mg subcutaneously every 8 weeks) within the study protocol of the clinical trial. In August 2018, he noticed painful swelling of the calves. Diagnostic workup showed no evidence of deep venous thrombosis or soft tissue infection. Retrospectively, the patient remembered a short episode of gastroenteritis at this time. Diuretic therapy resulted in temporary improvement, but in the following weeks, the patient’s symptoms worsened and spread to the upper extremity and proximal trunk muscles. Administration of systemic steroids and transient discontinuation of ibrutinib had no effect. In November 2018, the swelling of the patient’s calves worsened, but besides a single slightly enlarged lymph node of the right groin, no other new findings were present on physical examination. Laboratory tests showed elevated lactate dehydrogenase (LDH) and creatine kinase (CK) with a negative result of autoimmune serology. Analgesic treatment with metamizole and tilidine was initiated with limited success. In January 2019, a computed tomographic scan showed no signs of a lymphoma relapse but revealed diffuse subcutaneous edema. Continuous clinical deterioration was noted, with the patient being unable to walk properly. Swelling of the limbs progressed and eventually led to hospitalization. On admission, the patient was experiencing generalized muscle pain, and his performance score deteriorated to Eastern Cooperative Oncology Group 3 (ECOG 3). Massive generalized edema was present, especially of the lower extremities, accompanied by a slight erythema. Muscles of the trunk and the extremities were extremely palpation-sensitive and painful. Besides a weak symmetric fist closure and shoulder lift, no neurological deficit was apparent.

Initial findings were consistent with myositis of unknown cause. Differential diagnoses included autoimmune myositis either idiopathic or paraneoplastic due to undetected lymphoma relapse, therapy-related side effects, neurological disease or infectious disease such as lues or borreliosis. Because therapy-related side effects could not be ruled out, ibrutinib and rituximab were discontinued.

Initial laboratory testing revealed a distinct inflammatory constellation and slightly elevated transaminases (Table 1). The patient’s plasma protein and albumin levels as well as immunoglobulin G were decreased. His CK was elevated. Additional myositis panel testing did not indicate an autoimmune reaction, and analysis of bone marrow aspirate showed no evidence of lymphoma relapse. Further neurological diagnostics only confirmed mild neuropathy.

**Table 1:** Initial laboratory testing upon admission and at diagnosis of rhabdomyolysis and myocarditis

Value	Reference	18 Jan 2019	28 Jan 2019
Creatinine	0.7–1.3 mg/dl	0.86 mg/dl	2.14 (+)
eGFR	62–100 ml/minute/1.73 m^2^	96 ml/minute/1.73 m^2^	34 ml/minute/1.73 m^2^ (−)
Uric acid	3.5–7.2 mg/dl		10.9 mg/dl (+)
CRP	< 5 mg/L	58 mg/L (+)	59 mg/L (+)
CK	30–200 U/L	227 U/L (+)	470 U/L (+)
Troponin I	< 24 pg/ml		911 pg/ml (+)
Myoglobin	< 78 ng/ml		901.5 ng/ml (+)
LDH	245 U/L	586 U/L (+)	661 U/L (+)
ALT	< 50 U/L	54 U/L (+)	80 U/L (+)
AST	5–35 U/L	93 U/L (+)	167 U/L (+)
Plasma protein	64–83 g/L	45 g/L (−)	
Albumin	35–50 g/L	23 g/L (−)	
IgG	5.4–18.2 g/L	3,95 g/L (−)	
Leukocyte count	3.5–10/nl	11.3/nl (+)	17.8/nl (+)

(+) value increased; (−) value decreased

*Abbreviations: ALT* Alanine aminotransferase, *AST* Aspartate aminotransferase, *CK* Creatine kinase, *CRP* C-reactive protein, *eGFR* Estimated glomerular filtration rate, *IgG* Immunoglobulin G, *LDH* Lactate dehydrogenase

**Fig. 1:**
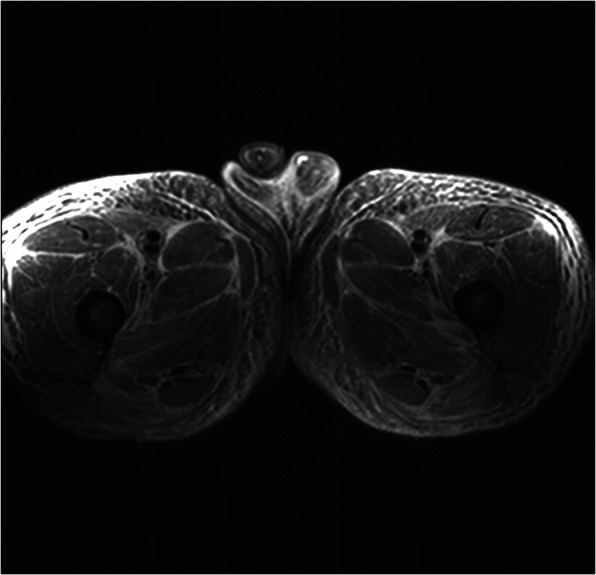
Magnetic resonance imaging (MRI) indicating myositis. Diffuse high intramuscular and extramuscular signals showing edema consistent with myositis (MRI short tau inversion recovery sequence, axial views)

**Fig. 2:**
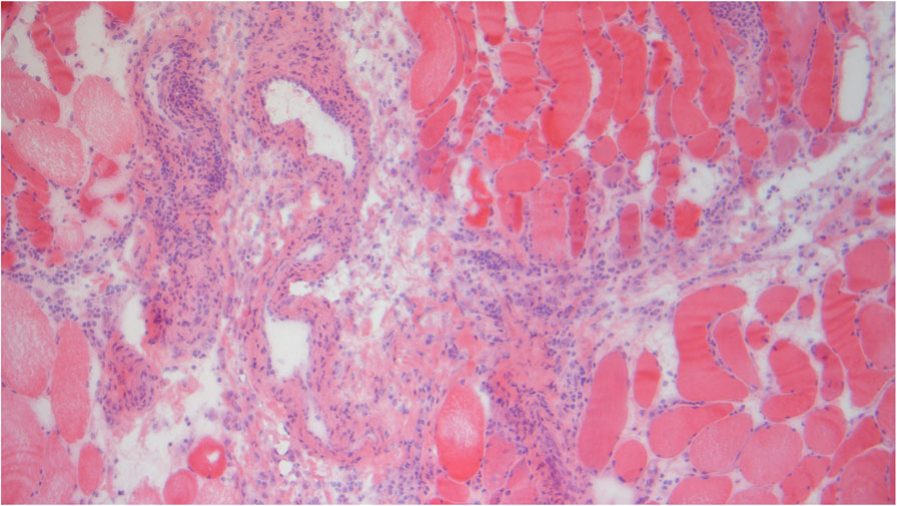
Biopsy revealed signs of viral myositis. Biopsy from the leg abductors showing vast interstitial and perivascular infiltrations by T cells and macrophages as well as the characteristic alterations in muscle fibers such as variation in size, centrally located nuclei, and stages of necrosis and regeneration (hematoxylin and eosin staining, original magnification × 100)

Magnetic resonance imaging (MRI) of the thighs was consistent with myositis (Fig. 1) and confirmed by muscle biopsy (Fig. 2). The patient rapidly developed acute kidney injury accompanied by further increase in uric acid, LDH, CK, leukocyte count, and myoglobin (Table 1). A diagnosis of rhabdomyolysis with consecutive kidney injury was made and treated symptomatically. At this time, a rise of troponin I level was noted, and electrocardiography (ECG) revealed a newly acquired right bundle branch block and disturbance of repolarization. Subsequently, the patient developed atrial fibrillation. Cardiac MRI confirmed the diagnosis of a myocarditis.

In parallel, increasing sleepiness and changes in personality became evident. MRI of the brain was inconspicuous, but a hyperintense lesion was detected in the spinal cord, which was highly suspicious for myelitis. Cerebrospinal fluid (CSF) analysis showed increased leukocyte counts as well as increased lactate and protein levels (Table 2). Extended microbiological and virological screening revealed a positive enterovirus polymerase chain reaction (PCR) in the CSF. Subsequently, enterovirus RNA was also found in the patient’s serum, stool samples, and muscle biopsy, whereas no antienterovirus antibodies were detectable in the serum. Further subtyping identified the virus as echovirus 11.

**Table 2:** Cerebrospinal fluid analysis

Value	Reference range	
Leukocyte count	< 5/μl	329/μl (+)
Lactate	1.7–2.6 mmol/L	3.5 mmol/L (+)
CSF protein	15–40 mg/dl	79.6 mg/dl (+)

(+): value increased

*CSF* Cerebrospinal fluid

Taken together, a multiorgan enterovirus infection with myositis, gastroenteritis, myocarditis, encephalitis, and hepatitis was diagnosed. Retrospectively, the gastroenteric symptoms in August 2018 are likely to indicate the primary enterovirus infection.

Treatment with high-dose intravenous immunoglobulin (IVIG; 2 g/kg body weight daily over 4 days followed by 0.5 g/kg once weekly) was initiated to restore humoral immunity. The clinical condition of the patient improved rapidly. His laboratory parameters and ECG normalized. One week after the first IVIG infusion, antienterovirus antibodies were detected in the serum. Moreover, enterovirus PCR in the patient’s CSF became negative, and the virus burden in his stool was reduced after 2 weeks. His neurologic symptoms also regressed, and he was discharged 6 weeks after admission.

At his latest follow-up visit, the patient’s clinical condition was found to have further improved to ECOG 1. Analgesic treatment could be reduced to low dosage with only occasional and mild pain attacks. The patient’s MCL was still in remission without any further antilymphoma treatment. IVIG treatment was continued at lower doses and with prolonged intervals.

## Discussion and conclusions

The concept of maintenance treatment or treatment until progression has entered oncological concepts for improving control of incurable cancers. In the lymphoma field, rituximab has been used for many years. In MCL, rituximab maintenance is frequently administered for 3 years and sometimes even longer. Ibrutinib, another B-cell-depleting agent, has become a relevant medication for MCL; it is typically applied as a single agent in relapsed or refractory disease. Infectious complications have been reported for each of both agents [2, 5, 6]. Ibrutinib is mostly associated with fungal and bacterial infections. However, serious viral infections also occur. BTK, the molecular target of ibrutinib, is a critical mediator in innate and adaptive immune response [7]. The suggested mechanisms leading to a higher susceptibility for infectious complications in patients treated with ibrutinib are inhibition of B-cell proliferation and impaired macrophage activation and phagocytosis [8, 9]. Also, some authors suggest off-target effects on non-BTK Tec family proteins relevant for adequate immune response [10].

Although synergistic toxicity of combined treatment with rituximab and ibrutinib has not been fully explored, aggravated immunosuppression can be expected. Although other reports have described single-organ manifestations of enterovirus infection associated with rituximab therapy [11–13], our patient’s case is characterized by an unprecedented multiorgan enterovirus disease, which is in line with a profound immunosuppression by a both functional and numerical B-cell depletion. Severe infectious causes are reported in various types of lymphoma but are more often associated with immunosuppressive therapy, especially antibodies against CD20 receptor such as rituximab or obinutuzumab [11, 14, 15]. Also, individuals with loss of BTK expression are at increased risk for enterovirus infections, although resistance to other viral infections is intact [16]. Dendritic cells of BTK-deficient patients showed impaired maturation and reduced production of certain interferons after *in vitro* stimulation with enterovirus but a normal response after stimulation with influenza virus [17]. The results suggest these specific deficiencies as one possible cause of severe enterovirus infections in BTK-deficient patients. It appears, therefore, that the combination of two agents known to specifically trigger serious enterovirus infections might have facilitated such a fulminant course of enterovirus infection in our patient.

There is no standard recommendation for the treatment of enterovirus infection. Fortunately, a severe course of the disease is rare even in immunodeficient patients [18]. Only a few small studies with highly heterogeneous cohorts of patients regarding virus subtypes, comorbidities, or clinical manifestations have been published [11, 19]. In addition, studies were mainly performed in pediatric patients [20, 21]. Treatment usually consisted of IVIG application, antiviral substances, or both. Outcomes ranged from complete recovery to death, with no patient- or virus-related characteristics being predictive of therapeutic success with IVIG or antiviral drugs. Among the latter, the capsid inhibitor pleconaril has been used in studies; however, current experience is limited [22, 23]. Echovirus 11 is associated with myocarditis in infants [24]. A specific treatment for this strain has not been established.

In our patient, IVIG treatment was successful with rapid clinical improvement, which is in line with earlier findings. However, the intensity and duration of treatment have not been defined. Treatment decisions are further complicated by the inability to reliably predict and determine the restoration of a protective immune response after cessation of immunosuppressive medication. In order to avoid a potential flare of the infection, we chose tapering of IVIG dosage rather than immediate discontinuation.

In summary, enterovirus infections in immunocompromised patients are a rare but potentially life-threating complication. The combination of rituximab with ibrutinib seems to deepen immunosuppression and might render patients more susceptible to multiorgan manifestations in cases of enterovirus infection, which needs to be considered and requires rigorous treatment. Ongoing combined use of ibrutinib and CD20 antibodies in future oncological therapy will show if these agents increase predisposition to this rare disease. High-dose IVIG therapy showed satisfying outcomes in several cases and should be considered as treatment for severe enterovirus infection. In severe enterovirus infections, we consider subtyping essential to identify strain differences in pathogenicity for the development of efficient preventive and therapeutic strategies.

## Supplementary information

**Additional file 1.** Timeline medical history.

**Additional file 2.** Timeline hospital course.

## Data Availability

Not applicable.

## Abbreviations

ALT: Alanine aminotransferase
AST: Aspartate aminotransferase
BTK: Bruton tyrosine kinase
CK: Creatine kinase
CRP: C-reactive protein
CSF: Cerebrospinal fluid
ECG: Electrocardiography
ECOG: Eastern Cooperative Oncology Group
eGFR: Estimated glomerular filtration rate
IgG: Immunoglobulin G
IVIG: Intravenous immunoglobulins
LDH: Lactate dehydrogenase
MCL: Mantle cell lymphoma
MRI: Magnetic resonance imaging
PCR: Polymerase chain reaction
RNA: Ribonucleic acid
