# Supplementary figures and images for: Life-threatening disseminated enterovirus infection during combined rituximab and ibrutinib maintenance treatment for mantle cell lymphoma: a case report
Source: J Med Case Rep. 2020 Aug 28;14:135. doi: 10.1186/s13256-020-02457-y (PMC7456041; doi:10.1186/s13256-020-02457-y)

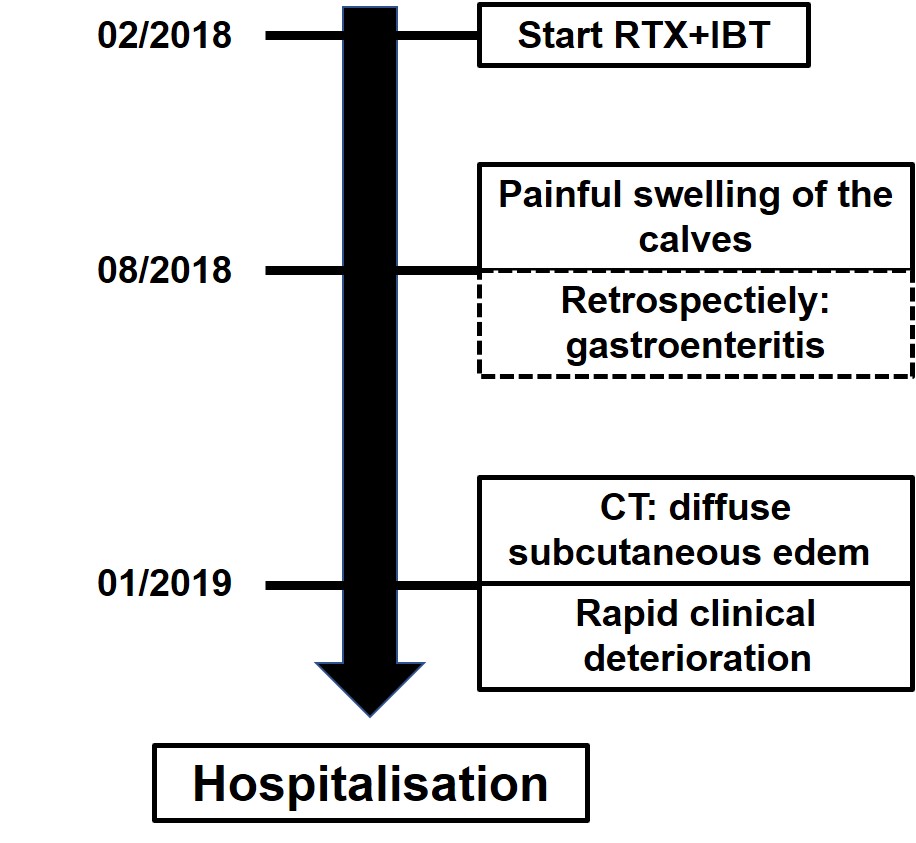

Supplement: Supplementary file 1 — Additional file 1. Timeline medical history. [file 13256_2020_2457_MOESM1_ESM.jpg]

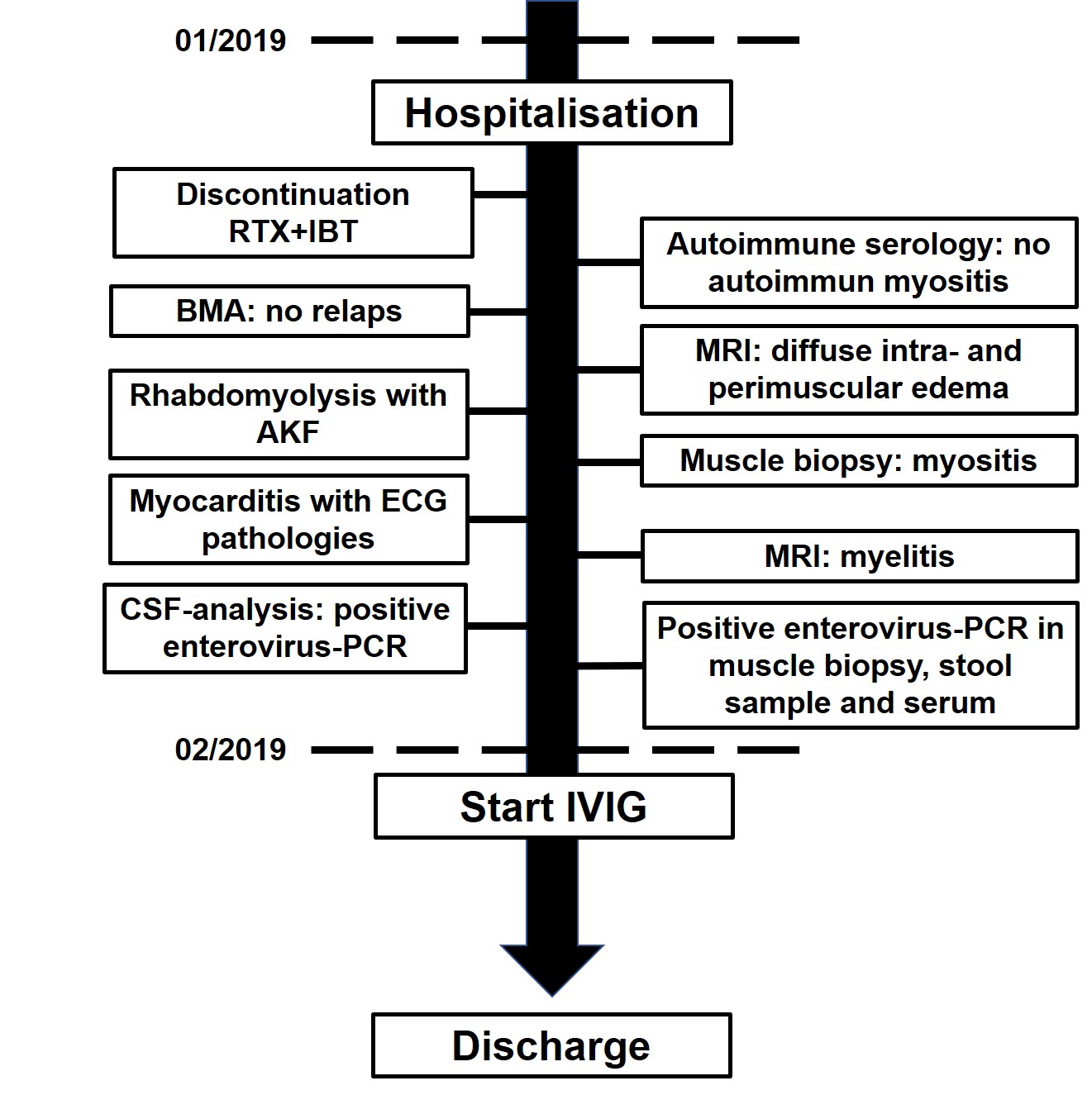

Supplement: Supplementary file 2 — Additional file 2. Timeline hospital course. [file 13256_2020_2457_MOESM2_ESM.jpg]
